# Motion-picture recording of ultrafast behavior of polarized light incident at Brewster’s angle

**DOI:** 10.1038/s41598-020-64714-w

**Published:** 2020-05-06

**Authors:** Mika Sasaki, Atsushi Matsunaka, Tomoyoshi Inoue, Kenzo Nishio, Yasuhiro Awatsuji

**Affiliations:** 1 Department of Electronics, Graduate School of Science and Technology, Kyoto Institute of Technology, Matsugasaki Goshokaido-cho, Sakyo-ku, Kyoto, 606-8585 Japan; 2 Advanced Technology Center, Kyoto Institute of Technology, Matsugasaki Goshokaido-cho, Sakyo-ku, Kyoto, 606-8585 Japan; 3 Faculty of Electrical Engineering and Electronics, Kyoto Institute of Technology, Matsugasaki Goshokaido-cho, Sakyo-ku, Kyoto, 606-8585 Japan

**Keywords:** Interference microscopy, Imaging and sensing

## Abstract

Observing light propagation plays an important role in clarifying ultrafast phenomena occurring on femtosecond to picosecond time scales. In particular, observing the ultrafast behavior of polarized light is useful for various fields. We have developed a technique based on Polarization Light-in-Flight Holography, which can record light propagation as a motion picture that can provide information about the polarization direction. Here we demonstrate motion-picture recording of a phenomenon, which is characteristic of polarized light, by using the proposed technique. As a phenomenon, we adopted the behavior of a light pulse incident at Brewster’s angle. We succeeded in recording the light reflection of specific polarized light by the proposed optical setup. The method of recording the motion-picture, reconstruction procedure, and the quantitative evaluation of the results are demonstrated.

## Introduction

Recently, applications of a pulsed laser operating in the picosecond or femtosecond regimes have been actively studied in various fields such as engineering^1–3^, physics^4,5^, and biology^6,7^. Observing the propagation of a light pulse is important for developing various studies in these fields. There have been many studies on the techniques for observing light pulse propagation. For example, femto-photography^8^, compressed ultrafast photography^9–11^, femtosecond time-resolved optical polarigraphy (FTOP)^12–15^, single-photon sensitive light-in-fight imaging^16,17^, pump-probe method^18–21^, light-in-flight (LIF) holography^22–28^, and so on. Velten et al. showed motion pictures of a propagating light pulse around the object using a streak camera^8^. The streak camera is an ultrafast photon detection system that transforms the temporal profile of the light signal into the temporal profile by pulling photoelectrons with a sweep voltage. However, a synchronization with scanning light source and repetitive light pulses were required to capture a two-dimensional (2D) image. To overcome this limitation, compressed ultrafast photography^9–11^ has been developed. The technique combines compressed sensing and a streak camera to capture motion pictures of light propagation without scanning procedure. Compressed ultrafast photography has been applied to various applications, including imaging of light pulse reflection and refraction, fluorescence lifetime imaging^9^, and real-time observing of a photonic Mach cone^10^. FTOP, based on the optical Kerr effect, can be used to observe the light pulse propagation^12–14^. However, FTOP requires repetitive light pulses, the profile of the light pulse propagation is different from shot to shot. Although a single-shot FTOP system^15^ was developed afterwards, the number of frames captured in one acquisition is limited by the trade-off between temporal resolution and the imaging field of view (FOV). Gariepy et al. used a SPADs (single-photon avalanche diodes) array of 32 pixels ×32 pixels to observe light propagation^16^. SPAD pixel offers extreme sensitivity and picosecond temporal resolution. The propagating light pulse in the air and optical fibers were observed by the SPADs^16,17^. The pump-probe technique is one of the most common methods to understand not only the ultrafast phenomena but also the light propagation. The light pulse propagation and terahertz Cherenkov wave generated by focusing femtosecond light pulse into lithium niobite (LiNbO_3_) were observed by using the pump-probe technique^20,21^. Compared with other techniques, LIF holography has the following advantages. First, it has a high temporal resolution. Second, it can record light propagation as a spatially and temporally continuous motion picture. Third, it can obtain three-dimensional images of a light pulse^27^. Moreover, we have developed a technique that can simultaneously obtain polarization information^28^, termed, Polarization Light-in-Flight Holography. Some ultrafast phenomena vary in their behavior depending on polarization state^29^, and some occur in polarization-sensitive materials^30^. Therefore, polarization properties are important for our increased understanding of such phenomena.

In this paper, we report motion-picture recording of a phenomenon that is characteristic of polarized light by using the Polarization Light-in-Flight Holography. As a subject, we chose a light pulse incident at Brewster’s angle. The phenomenon of Brewster’s angle is well-known, which was discovered about two hundred years ago^31,32^. However, to the best of our knowledge, no one has observed the reflection of specific polarized light as a motion picture. Our experimental results are the first demonstration that the phenomenon is observed as motion pictures, instead of still pictures of the trajectory of the light rays. Moreover, we quantitatively evaluate the result obtained by the Polarization Light-in-Flight Holography for the first time.

## Experimental Results

A short-pulsed laser is used as a light source in the recording procedure of the Polarization Light-in-Flight Holography. Different horizontal points on the holographic plate get the information about the subject (light pulse) generated at different times. On the other hand, different longitudinal areas on the holographic plate get information about polarization. In order to obtain the polarization information, we record the amounts of the four polarization components included in the subject. This is achieved by the interference between the subject (object light pulse) and one of the four reference light pulses that vary in polarization direction. Therefore, four motion pictures, characterizing each polarization component, are recorded. The amounts of the four polarization components correspond to the intensities of the four reconstructed images. The detailed method and experimental setup are described in the next sections.

**Figure 1:**
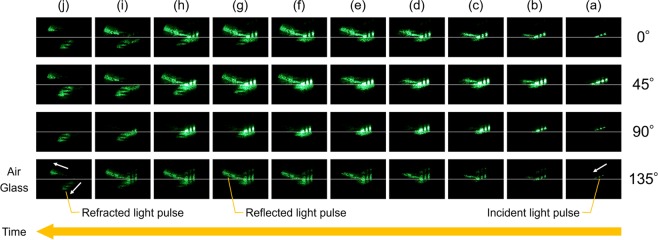
Experimental results. The white arrows indicate the motion direction. We capture the different motion pictures simultaneously (Video S1). In addition, we separately show individual motion pictures as other video files (Video S2, Video S3, Video S4, and Video S5). (**a**) The pulse is in air. (**b**) The pulse has reached the glass and the reflected light pulse has appeared. (**c**–**j)** Propagation of the reflected light pulse can be observed. (**e**–**j**) Propagation of the refracted light pulse can be observed.

Figure 1 shows the experimental results. They are the reconstructed images extracted from the recorded motion pictures at different time with different polarization components (see Video S1, Video S2, Video S3, Video S4, and Video S5). The numbers (0°-135°) on the right-hand side in Fig. 1 indicate each polarization component. The white lines show the air-glass interface. The recording time and the time interval between each image are 290 fs and 30 fs, respectively. The black lines shown in the reconstructed images indicate a background pattern. It was used for evaluating the magnification. It is observed that there is a little change between the 5th and 6th frames, as depicted in Fig. 1(e,f). However, there are remarkable changes between the 1st and 3rd frames and between the 8th and 10th frames, as depicted in Fig. 1(a–c,h–j).

Four reconstructed images, recorded at the same time, vary in intensity according to the polarization components. For example, regarding the incident light pulse, the reconstructed intensity of an image of the 45° linearly polarized component is the highest and that of the 135° linearly polarized component is the lowest, as depicted in Fig. 1. Moreover, regarding the reflected light pulse, the reconstructed image intensity of the 0° linearly polarized component is the highest, and that of the 90° linearly polarized component is too low to observe. From these results, it can be inferred that the polarization information of the light pulse incident at Brewster’s angle can be obtained successfully.

## Discussion

**Figure 2:**
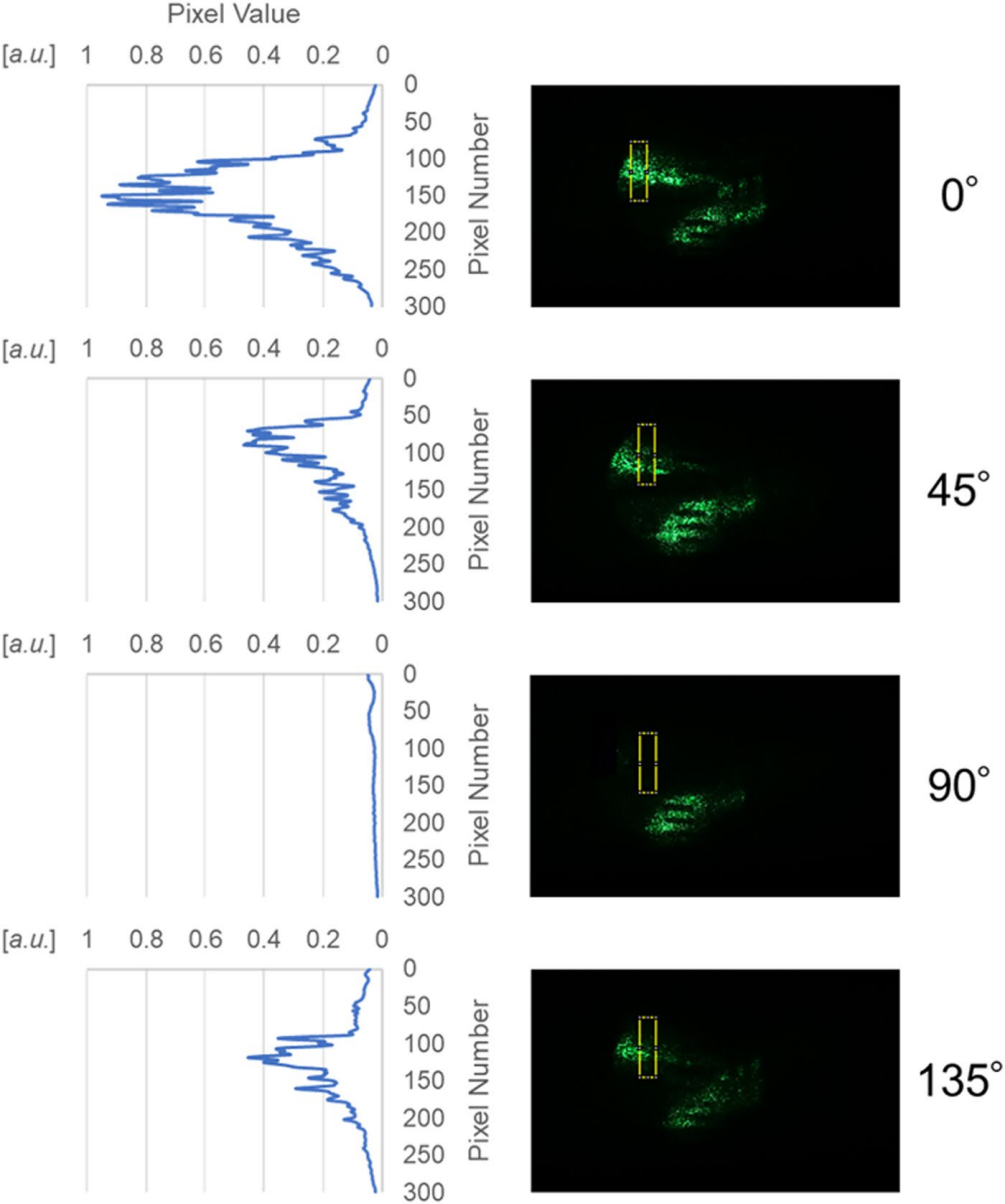
Standardized pixel values of the images of the reflected light pulse.

We evaluated the reconstructed image intensity obtained in the present study. Figure 2 shows the standardized pixel values of the images of the reflected light pulse. The numbers (0°-135°) on the right-hand side, in Fig. 2, indicate each polarization component. The yellow rectangles indicate the measuring areas, and their size is 300 pixels × 80 pixels. The standardized pixel values of the 0°, 45°, 90°, and 135° linearly polarized components at their maximum are 0.93, 0.47, 0.047, and 0.46, respectively.

We quantitatively discussed the dependence of the reconstructed image intensity on polarization. Here, we defined the difference angle in polarization direction between the object light pulse and the reference light pulse as $\alpha$. In conclusion, the relationship between the pixel values of the four reconstructed images and $\alpha$ is not linear. Because of the Malus’s law^33^, the pixel value of the reconstructed image depends on ${{\rm{\cos }}}^{2}(\alpha )$. Assuming that the pixel value of the reconstructed image at $\alpha =0^\circ$ is 1 and the pixel value of the reconstructed image at $\alpha =45^\circ$ is 0.5. From the results of Fig. 2, it can be shown quantitatively that 0° polarized light selectively appears as reflected light, and this result matches the experimental condition.

## Method

The Polarization Light-in-Flight Holography (the Polarization LIF Holography) is one of the ultrafast imaging techniques, from which it is possible to obtain motion pictures with polarization information of a propagating light pulse. It contains two-component techniques. One technique enables us to record light pulse propagation as a spatially and temporally continuous motion picture. The principle of this technique is the same as the usual LIF holography. The other enables us to visualize the polarization direction of a propagating light pulse. In order to identify polarization direction, we must obtain the intensity images of different polarized components. One of the powerful polarization imaging tools, the polarization camera^34,35^, generally captures intensity images of four linear polarized components; therefore, we adopted the method of acquiring the four intensity images.

**Figure 3:**
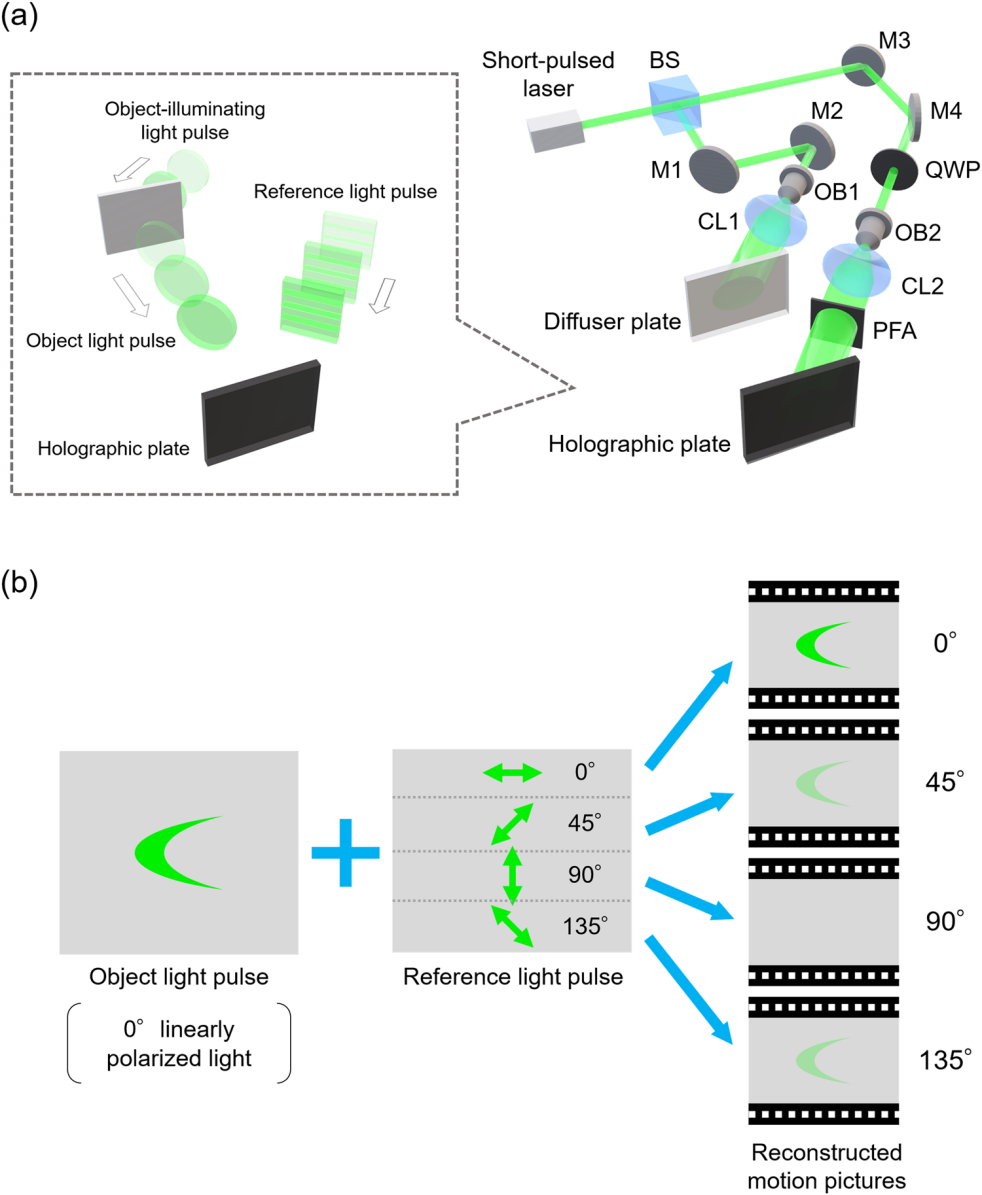
Schematic of the Polarization Light-in-Flight Holography. (**a**) Optical setup for the technique. M1-M4, mirrors; BS, beam splitter; QWP, quarter-wave plate; OB1 and OB2, objective lenses; CL1 and CL2, collimator lenses; and PFA, polarizing filter array. (**b**) The basic concept of the technique to visualize the polarization direction of a propagating light pulse.

First, we explain the principle of the first component technique to record light pulse propagation as a spatially and temporally continuous motion picture. Figure 3(a) shows an optical setup of the Polarization LIF Holography. In the recording procedure, a short-pulsed laser is used as a light source. A light pulse emitted from the short-pulsed laser is divided into two light pulses by a beam splitter. One light pulse illuminates the object and it is called the object-illuminating light pulse. The other is called the reference light pulse. The object-illuminating light pulse is obliquely introduced to a diffuser plate with a certain incident angle. Then, the object-illuminating light pulse sweeps different horizontal points on the diffuser plate. The object-illuminating light pulse is scattered by the diffuser plate, then light pulses are generated from different points at different times. These light pulses are called the object light pulses. The reference light pulse is also obliquely introduced to a holographic plate with a certain incident angle, $\theta$, and then sweeps different horizontal points on the holographic plate. The interference between the object light pulses and the reference light pulse occurs only when they arrive at a point on the holographic plate simultaneously. In other words, the different horizontal points on the holographic plate get the information about an object light pulse generated at different times. In the reconstruction procedure, a continuous wave (CW) laser is used for reconstructing the motion picture of the light pulse propagation. We choose a CW laser emits light whose wavelength is approximately the same as the center wavelength of the pulsed laser used to record the interference pattern or the holograms. The light emitted from the CW laser is collimated and illuminates the hologram at the angle, $\theta$. By moving the gazed point on the hologram along the direction in which the reference light pulse swept the holographic plate, we can observe an optical image of a spatially and temporally continuous motion picture of the light pulse propagation.

Next, we explain the principle of the second component technique, i.e., visualization of the polarization direction of a propagating light pulse. The basic concept of the technique is one of the Fresnel-Arago Laws^36^ stating that two orthogonal, coherent linearly polarized waves cannot interfere. A polarizing filter array (PFA) is introduced into the reference light pulse path. PFA is made by longitudinally arranging four pieces of linear polarizing film whose respective transmission axes are 0°, 45°, 90°, and 135°. The reference light pulse is changed to circularly polarized light pulse by a quarter-wave plate (QWP) before PFA. Thus, PFA gives longitudinal spatial distribution of four linear polarized light pulses to the reference light pulse. The holographic plate is divided longitudinally, and then we obtain four holograms that vary in the polarization direction of the reference light pulse. For example, we obtain four motion pictures, as shown in Fig. 3(b), when we record the propagation of a 0° linearly polarized light pulse. The object light pulses and the 0° linearly polarized reference light pulse interfere most constructively. However, the object light pulses and the 90° linearly polarized reference light pulse do not interfere. The contrast of the interference fringes corresponds to the reconstructed image intensity. Thus, the reconstructed image intensity of the 0° linearly polarized component is the highest of the four images. On the other hand, the reconstructed image intensity of the 90° linearly polarized component is the lowest. The intensities of the reconstructed images of the 45° and 135° linearly polarized components are intermediate because the 45° and 135° linearly polarized reference light pulses include the 0° linearly polarized component. This is why we can obtain the polarization information of the object light pulse from the four reconstructed images.

## Experimental Setup

**Figure 4:**
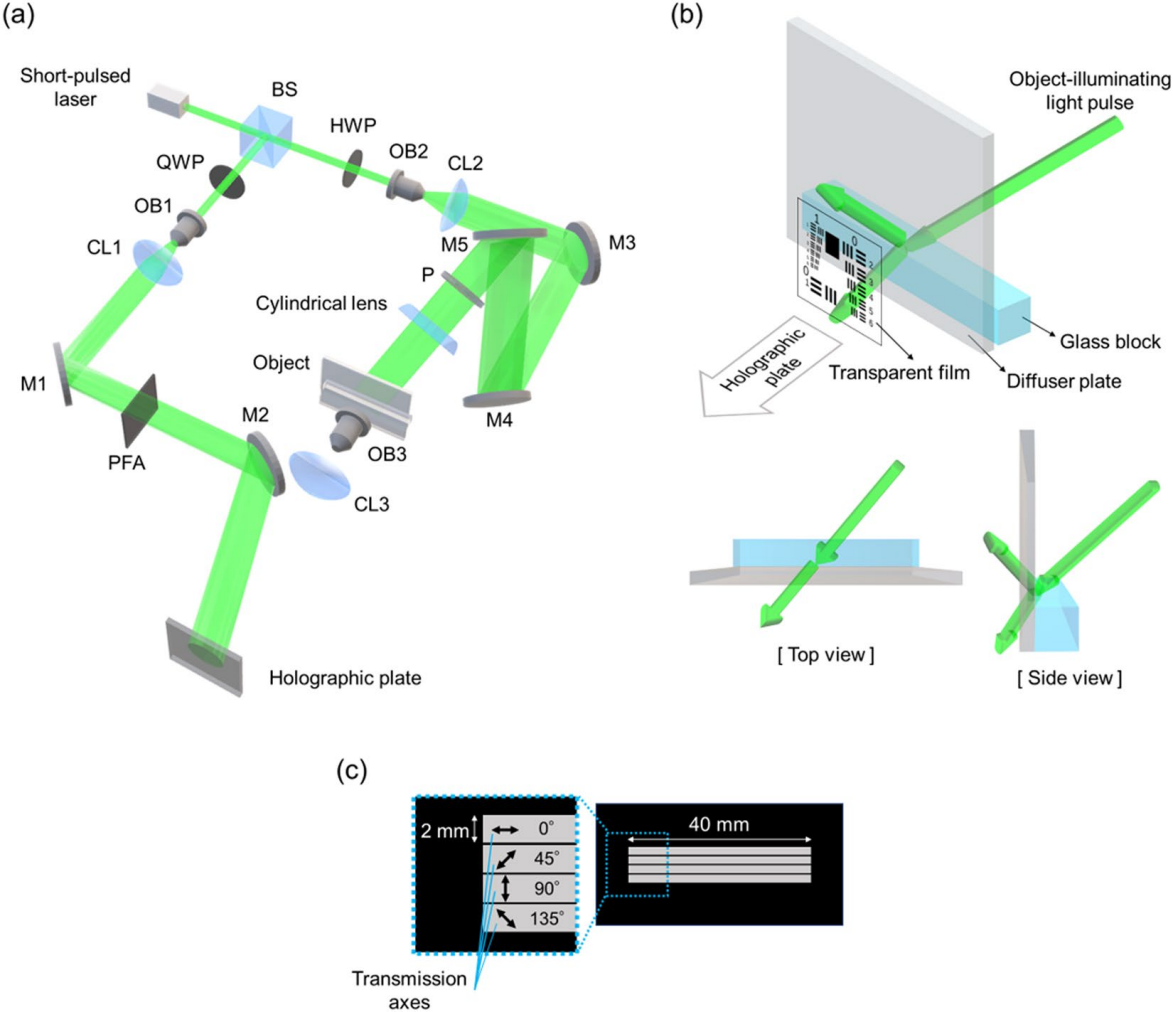
Experimental setup. (**a**) Optical setup for the experiment. M1-M5, mirrors; BS, beam splitter; QWP, quarter-wave plate; HWP, half-wave plate; OB1-OB3, objective lenses; CL1-CL3, collimator lenses; P, polarizer; and PFA, polarizing filter array. (**b**) Detail view of the object. (**c**) PFA, the polarizing filter array.

Figure 4(a)shows the experimental setup. A mode-locked pulsed laser (HighQ-2 SHG, Spectra-Physics Inc.) was used for the light source. The pulse duration and the central wavelength were 178 fs and 522 nm, respectively. A Konica P-5600 was used as the recording medium of the interference (hologram). As shown in Fig. 4(b), the object-illuminating light pulse illuminated the object that was made with a diffuser plate, a glass block, and a transparent film. A USAF test target was printed on the film. The film was not set to evaluate the resolving power of this system but to notice the light pulse propagation easily. As shown in Fig. 4(c), PFA was made with four polarizing films, and the size of each was 2 mm × 40 mm. In order to observe the air-glass interface in detail, we placed a magnifying optical system into the object light pulse path. The object-illuminating light pulse and the reference light pulse were introduced from the opposite direction because images of the object light pulses were reversed up/down and left/right by the magnifying optical system. The object-illuminating light pulse was reflected diagonally upward by the mirror, M4 and it was incident on the upper-end surface of the glass block at Brewster’s angle by the mirror, M5. The longitudinal section of the light pulse was thick because the object-illuminating light pulse was incident from the obliquely upward direction. When the longitudinal section of the light pulse is thick, it is not easy to observe light propagation. Therefore, a cylindrical lens was placed into the path of the object-illuminating light pulse in order to observe the shape of the light pulse clearly. By using a half-wave plate and a polarizer, the incident light pulse was adjusted to a 45° linearly polarized light pulse consisting of s-polarized and p-polarized components equally. The purpose of the adjustment is to record different behavior of differently polarized light. A Nd:YVO_4_ laser emitting a CW light beam, whose wavelength is 532 nm, was used to reconstruct the motion picture. Thanks to the setup, we succeeded in observing the behavior of the light pulse incident at Brewster’s angle as motion pictures, for the world’s first time.

## Supplementary information

Video S1.

Video S2.

Video S3.

Video S4.

Video S5.
